# Evaluation of accelerated motion-compensated 3d water/fat late gadolinium enhanced MR for atrial wall imaging

**DOI:** 10.1007/s10334-021-00935-y

**Published:** 2021-06-24

**Authors:** Camila Munoz, Iain Sim, Radhouene Neji, Karl P. Kunze, Pier-Giorgio Masci, Michaela Schmidt, Mark O’Neill, Steven Williams, René M. Botnar, Claudia Prieto

**Affiliations:** 1 School of Biomedical Engineering and Imaging Sciences, St Thomas’ Hospital, King’s College London, 3rd Floor, Lambeth Wing, London, SE1 7EH UK; 2 MR Research Collaborations, Siemens Healthcare Limited, Frimley, UK; 3 Cardiovascular MR Predevelopment, Siemens Healthcare GmbH, Erlangen, Germany; 4 Escuela de Ingeniería, Pontificia Universidad Católica de Chile, Santiago, Chile

**Keywords:** 3D atrial LGE, Water/fat LGE, Respiratory motion-correction

## Abstract

**Objective:**

3D late gadolinium enhancement (LGE) imaging is a promising non-invasive technique for the assessment of atrial fibrosis. However, current techniques result in prolonged and unpredictable scan times and high rates of non-diagnostic images. The purpose of this study was to compare the performance of a recently proposed accelerated respiratory motion-compensated 3D water/fat LGE technique with conventional 3D LGE for atrial wall imaging.

**Materials and methods:**

18 patients (age: 55.7±17.1 years) with atrial fibrillation underwent conventional diaphragmatic navigator gated inversion recovery (IR)-prepared 3D LGE (dNAV) and proposed image-navigator motion-corrected water/fat IR-prepared 3D LGE (iNAV) imaging. Images were assessed for image quality and presence of fibrosis by three expert observers. The scan time for both techniques was recorded.

**Results:**

Image quality scores were improved with the proposed compared to the conventional method (iNAV: 3.1 ± 1.0 vs. dNAV: 2.6 ± 1.0, *p* = 0.0012, with 1: Non-diagnostic to 4: Full diagnostic). Furthermore, scan time for the proposed method was significantly shorter with a 59% reduction is scan time (4.5 ± 1.2 min vs. 10.9 ± 3.9 min, *p* < 0.0001). The images acquired with the proposed method were deemed as inconclusive less frequently than the conventional images (expert 1/expert 2: 4/7 dNAV and 2/4 iNAV images inconclusive).

**Discussion:**

The motion-compensated water/fat LGE method enables atrial wall imaging with diagnostic quality comparable to the current conventional approach with a significantly shorter scan of about 5 min.

## Introduction

Late gadolinium enhanced (LGE) cardiac magnetic resonance (MR) imaging is a promising non-invasive tool for the comprehensive assessment of atrial morphology and fibrosis in patients with atrial fibrillation [1–3]. Several studies have demonstrated that the presence of fibrosis detected by LGE in the atrial wall can predict clinical outcome in patients before atrial ablation and can therefore be used for patient management and therapy planning [2, 4–6]. Furthermore, LGE cardiac MR has been used for the quantification of post-ablation scar, including assessment of therapy delivery and potential repeat ablation [1, 7–9].

Most atrial LGE imaging protocols are currently based on free-breathing diaphragmatic navigator-gated (dNAV) inversion recovery (IR)-prepared 3D gradient echo sequences, which enable the acquisition of images with sufficient spatial resolution (~ 1.3 mm in plane, 3-4 mm slice thickness) and volumetric coverage for an accurate depiction of the thin atrial wall. Data are typically acquired using a subject-specific inversion time (TI) which nulls the signal arising from the healthy myocardium, starting ~ 15–25 min after injection of a Gadolinium-based contrast agent, which allows for washout of the contrast agent in the blood and therefore improves fibrosis-to-blood pool contrast.

Although these sequences can produce diagnostic quality images in subjects with regular breathing patterns, low respiratory efficiency in subjects with irregular breathing often lead to poor image quality, due to residual motion artefacts and/or signal variations arising from heart rate variability and contrast agent washout during long scans [10, 11]. In addition, the use of dNAVs for respiratory motion compensation may result in inflow artefacts that are usually observed as a high signal intensity in the pulmonary veins, which can obscure the detection of fibrosis in that region [12]. Finally, while gadolinium dose and imaging timing protocols have been optimized to maximize the contrast between atrial LGE and atrial blood pool while nulling signal arising from healthy atrial wall, prolonged and unpredictable acquisition times of up to 15 min result in further image quality degradation due to gadolinium washout during the scan. Overall, an average ~ 23% of atrial LGE scans are reported as non-diagnostic in several studies (range 13.5–40.6% [2, 4, 5, 7, 10, 13, 14]), which in addition to the time-consuming protocol required for atrial wall LGE imaging has hindered its wide adoption in the clinical routine.

Some technical developments have focused on reducing the inflow artefact in the pulmonary veins, produced by the navigator restore pulse that is performed immediately after the IR pulse to restore liver signal, by either replacing the dNAV with an external device, such as abdominal bellows, for respiratory gating [12], or by modifying delays between preparation pulses in the acquisition sequence [15, 16]. Although by using external devices the inflow artefact is completely removed, these devices only measure the respiratory-induced motion of the heart indirectly. Indeed, the relationship between the motion of the heart and the bellows signal is non-linear, hindering their use for advanced motion compensation techniques that require quantitative motion signals [17]. Furthermore, water/fat LGE imaging has been shown to produce atrial LGE images with a more homogeneous blood pool signal compared to conventional fat saturation, while simultaneously reducing inflow artefacts [18]. However, all of these approaches use a conventional prospective respiratory gating model for motion compensation, where data are accepted for image reconstruction only when the respiratory signal is within a narrow respiratory window (usually end-expiration), resulting in long and unpredictable scan times.

In order to reduce scan time, techniques that employ radial stack-of-stars acquisitions and respiratory-resolved image reconstruction have been recently proposed [19]. While this approach enables atrial LGE imaging with good spatial resolution (1.5 × 1.5 × 2 mm) with a shorter and predictable scan time of about 6 min, the reconstruction algorithm requires over 60 min of processing time, which is impractical for wide adoption in the clinical routine.

Recently, a novel 3D water/fat sequence that enables free-breathing LGE imaging in an overall short scan time with 100% respiratory scan efficiency (no data rejection) and undersampled acquisition has been introduced [20, 21]. This sequence replaces the conventional dNAV including the navigator restore pulse with 2D image navigators (iNAVs) [22], which allow the tracking of the respiratory position of the heart on a beat-to-beat basis, so that respiratory motion estimated from the iNAVs and from the 3D data itself can be integrated into the image reconstruction algorithm to produce motion-compensated images without the need to reject any of the acquired data. The sequence uses a dual-echo readout in order to enable water/fat separation, improving fat suppression in the LGE images and producing a complementary image of the fat distribution. Furthermore, the sequence utilizes an undersampled Cartesian variable-density spiral-like trajectory [23] with favorable undersampling properties to enable further acceleration of data acquisition. This sequence has been shown to produce good image quality for the depiction of left ventricular myocardial scar, however, its performance for atrial wall imaging has not been studied so far.

The aim of this work was to study the feasibility of using this accelerated motion-compensated water/fat sequence for efficient atrial 3D LGE imaging and characterize its performance in terms of image quality and overall acquisition time compared to conventional atrial wall imaging.

## Materials and methods

### Patient population

Eighteen patients (age: 55.7 ± 17.1 years, 16 male, 2 female) who were referred for a clinical cardiac MR examination for atrial substrate characterization were recruited for this study between July 2019 and February 2020. Patients were eligible to participate if they were > 18 years of age and agreed to 15 min of additional MR imaging after the routine clinical imaging protocol. The cohort included patients scheduled for either first time atrial fibrillation ablation or post-ablation follow-up, and their demographics are summarized in Table 1. The study was performed in accordance with the Declaration of Helsinki and approved by the National Research Ethics Service (REC 15/NS/0030). Written informed consent was obtained from each participant according to institutional guidelines.

**Table 1:** Summary of patient demographics

	Total (*n* = 18)
Age (years)	55.7±17.1
Gender	16 M, 2F
Type of AF	9 persistent, 7 paroxysmal, 1 ARVC-induced
Ablation status	14 pre-ablation, 4 post-ablation
AF duration (years)	3.8±4.7

*AF* atrial fibrillation; *ARVC* Arrhythmogenic right ventricular cardiomyopathy

### Accelerated motion-compensated water/fat MR imaging sequence

**Fig. 1:**
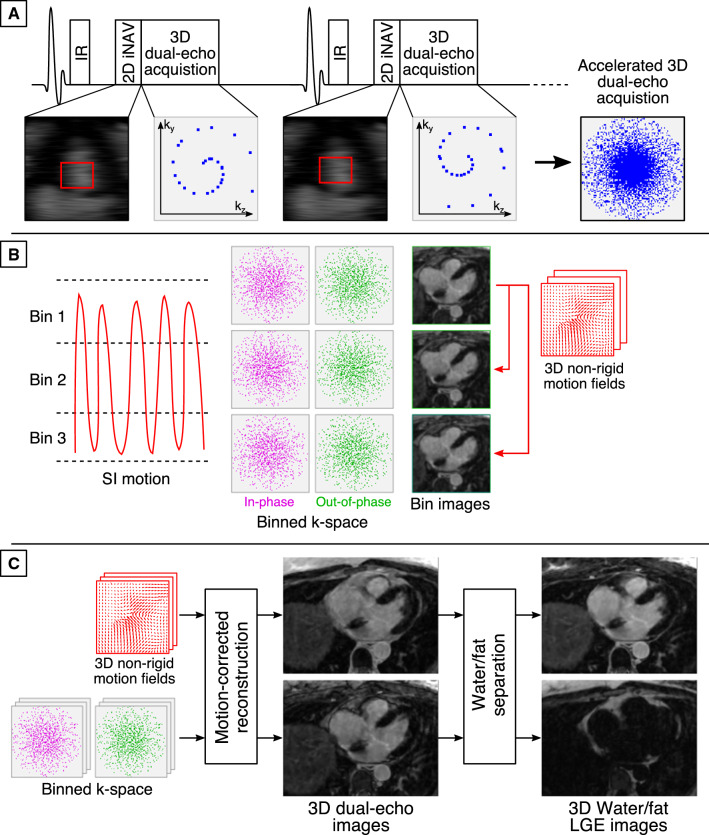
Overview of the accelerated motion-compensated 3D iNAV-based water/fat LGE imaging approach. **A** 2D iNAVs are acquired before 3D dual-echo undersampled data acquisition and used to estimate superior-inferior (SI) and right-left (RL) translational respiratory motion of the heart by tracking a template located around the atria (red rectangle). **B** SI motion is used to group the 3D data in a set of respiratory bins, and SI and RL motion estimates are used to correct intra-bin translational motion. Images reconstructed at each respiratory position are then used to estimate non-rigid deformation fields. **C** The binned data and motion fields are used in a motion-corrected reconstruction framework and the resulting motion-corrected dual-echo 3D images are then used to compute the final water/fat 3D LGE images

The accelerated motion-compensated MR image acquisition sequence consists of an ECG-triggered dual-echo 3D inversion recovery (IR)-prepared spoiled gradient echo sequence (Fig. 1 A), which enables water/fat 3D LGE imaging as described in [20]. Briefly, 3D dual-echo data are acquired with an undersampled variable-density golden-step Cartesian trajectory with spiral profile order sampling [23, 24], so that one spiral interleaf is acquired for each IR preparation pulse. Low-resolution coronal 2D iNAVs [22, 25] are acquired before the acquisition of 3D data, and are used to estimate the respiratory position of the heart in the superior-inferior (SI) and right-left (RL) directions by tracking a template located around the left atria.

The respiratory motion estimates from the 2D iNAVs are then used to produce motion-compensated 3D dual-echo datasets (Fig. 1B). Briefly, the SI motion is used to group the acquired 3D dual-echo data into a number of equally populated respiratory windows (or bins). For each bin, 3D dual-echo data are respiratory motion corrected to the center of the bin using corresponding SI & RL motion estimates. Respiratory-resolved images are then reconstructed using a soft-binning approach [26, 27] using iterative SENSE, so that 3D dual-echo images are obtained at each respiratory position. Bin-to-bin respiratory deformation fields are then estimated by performing non-rigid registration of 3D out-of-phase images [28], using the end expiratory bin as reference position. These non-rigid deformation fields are incorporated into a generalized matrix formulation for motion-corrected MR reconstruction [29], so that at the end of the image reconstruction process non-rigid motion-corrected 3D dual-echo images are obtained. Finally, these images are used as input for a 2-point Dixon algorithm to produce the final water/fat 3D LGE images [30] (Fig. 1C). The described motion-compensated reconstruction including respiratory binning and non-rigid motion correction was performed in-line on the scanner software.

### Experiments

All acquisitions were performed on a 1.5 T MRI system (MAGNETOM Aera, Siemens Healthcare, Erlangen, Germany) using an 18-channel chest-coil and a 32-channel spine coil. The clinical protocol included conventional dNAV-based 3D LGE imaging, with acquisition starting 20 min after bolus injection of a double dose (0.2 mmol/kg) of a Gd-based contrast agent (Gadovist, Bayer, Berlin, Germany), with a fat-suppressed IR-prepared sequence. Relevant dNAV-based 3D LGE imaging parameters include: transverse orientation, voxel size 1.3 × 1.3 × 4 mm^3^, interpolated to 1.3 × 1.3 × 2 mm^3^ during image reconstruction, TR/TE = 3.52/1.74 ms, flip angle = 20°, bandwidth = 365 Hz/px, parallel imaging (GRAPPA with twofold undersampling and 24 reference lines), navigator gating acceptance window of size 5 mm in end-expiration.

After conventional dNAV 3D LGE imaging, data were acquired with a prototype implementation of the iNAV water/fat 3D LGE imaging sequence without additional administration of contrast agent. iNAV water/fat 3D LGE data was acquired with matching transverse orientation, voxel size and field of view, and the following parameters: TR/TE1/TE2 = 7.16/2.38/4.76 ms, flip angle = 20°, bandwidth 495 Hz/px and threefold undersampling with elliptical shutter.

For both acquisitions (i.e., dNAV and water/fat iNAV 3D LGE) a subject-specific trigger delay (275–400 ms) and acquisition window (60–110 ms) were set to coincide with atrial mid-diastole in order to minimize the effect of cardiac motion. Both of these parameters were estimated by visually inspecting a conventional breath-held 4-chamber cine acquisition. In order to enable sufficient time for signal recovery after the IR preparation pulse while maintaining a clinically feasible scan time, data was acquired every heartbeat in patients with a heart rate < 70 bpm, and every other heartbeat otherwise (identical for both dNAV and iNAV 3D LGE). The inversion time was selected to null the signal from viable myocardium by visually inspecting a breath-held 4-chamber 2D TI scout Look-Locker image acquired immediately before each 3D LGE acquisition that matched the triggering scheme of the 3D acquisition (i.e., every heartbeat for patients with heart rate < 70 bpm, and every other heartbeat otherwise).

### Data analysis

Overall image quality and detectability of fibrosis in the atrial wall were analyzed for the conventional dNAV 3D LGE images and the iNAV 3D LGE water images. Qualitative grading of the images was performed by three cardiologists with > 3, > 10, and > 14 years of experience in cardiac MR imaging (I.S., S.W., P.G.M, respectively), who were blinded to patient information and history. Detectability of fibrosis was performed by two of the experts separately (I.S., S.W.) with > 3 and > 10 years of experience in atrial cardiac MR imaging and atrial fibrillation. Both dNAV 3D LGE images and iNAV 3D LGE water images were imported to Osirix (Bernex, Switzerland) for assessment, so that brightness/contrast could be freely adjusted.

For each dataset, presence of fibrosis in the atrial wall was assessed with 3-class criteria: Absent with confidence, Present with confidence, or Inconclusive. Furthermore, all the images were graded in terms of image quality using a 4-point scale, with 1: Non-diagnostic, 2: Acceptable, 3: Good, 4: Full diagnostic. This assessment considered the following criteria: ability to visualize fibrosis, presence of inflow artefacts, respiratory motion induced artefacts and/or blurring, nulling point of the viable myocardium and quality of fat suppression. Image quality scores for each expert were compared with a paired Wilcoxon signed-rank test to assess statistical differences; *p* < 0.05 was considered statistically significant. Image quality scores provided by each expert reviewer are presented separately, and the average score for each method is reported as a summary statistic. Krippendorff’s alpha coefficient was computed to assess inter-rater reliability. In this study, agreement was interpreted as follows: alpha < 0.677 = inconclusive, 0.677–0.80 tentative inter-rater reliability, 0.80–1.00 high inter-rater reliability [31].

Time between administration of Gd-based contrast agent and start of each 3D LGE acquisition, and acquisition time were recorded for all scans. Acquisition time for both sequences were compared with a paired 2-tailed Student *t* test to assess statistical differences, with *p* < 0.05 considered statistically significant. All statistical analysis was performed using the software package SPSS (Version 26.0.0.1). Finally, in order to visually compare atrial fibrosis depiction for each patient, atrial shell images were generated for both the dNAV and iNAV 3D LGE images using the open source software CEMRG [32].

## Results

The water/fat iNAV 3D LGE acquisition was successfully completed in all eighteen subjects with an average scan time of 4.5 ± 1.2 min, which was significantly shorter than the time required for conventional dNAV 3D LGE imaging (10.9 ± 3.9 min, *p* < 0.001). The heart rate of the subjects during the dNAV 3D LGE scan was 76.4 ± 14.2 bpm, while during the water/fat iNAV 3D LGE scan was 78.4 ± 14.5 bpm, with no statistically significant difference observed between both scans (*p* = 0.15).

**Fig. 2:**
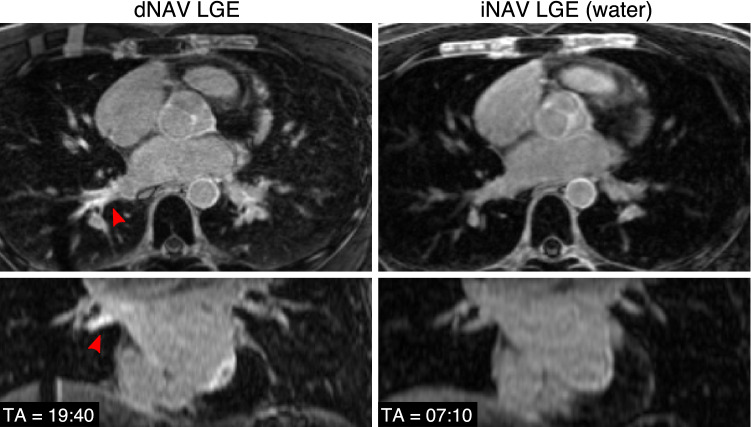
Visual comparison of 3D LGE images acquired with the conventional dNAV-based approach and the iNAV-based approach for a representative patient without LGE findings. iNAV-based 3D LGE reduces inflow artefacts in the pulmonary vein present in the dNAV images (red arrow). Acquisition times (TA) are expressed as min:s

**Fig. 3:**
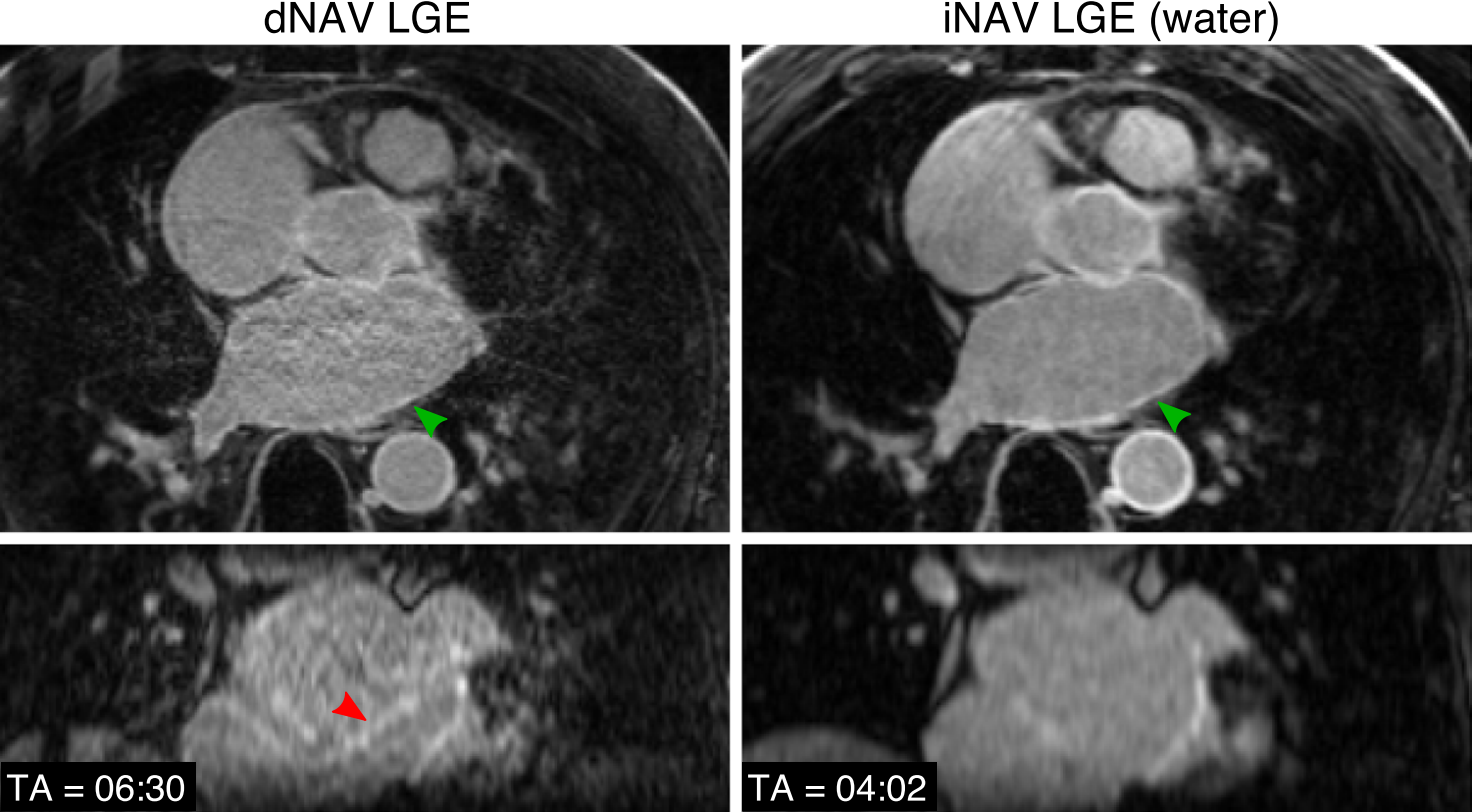
Visual comparison of 3D LGE images acquired with the conventional dNAV-based approach and the iNAV-based approach for a representative patient with observable atrial fibrosis (green arrows). Motion-induced artefacts of intensity similar to the LGE findings can be observed in the dNAV-based images (red arrow). Acquisition times (TA) are expressed as min:s

Representative images obtained with both dNAV- and iNAV-based approaches for a patient without LGE findings are shown in Fig. 2, including an axial and coronal view. While a comparable depiction of the atrial wall is obtained with both techniques, an apparent inflow artefact can be observed in the dNAV-based image (red arrows), which is not present in the iNAV-based image. Figure 3 shows a similar visual comparison for a patient with atrial fibrosis findings (green arrows). Motion-induced artefacts can be observed in the dNAV-based image which are of similar intensity to the LGE areas and could potentially be misinterpreted as fibrosis, whereas the iNAV-based approach does not present evident remaining respiratory motion artefacts.

**Fig. 4:**
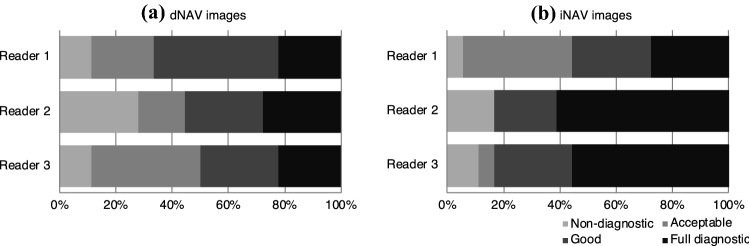
Image quality scores for (**a**) dNAV-based and (**b**) iNAV-based images for three expert readers. Average score for dNAV-based images was 2.6 ± 1.0 while for iNAV-based images was 3.1 ± 1.0. For reader 1 there was no statistically significant difference between methods (*p* = 1), while for readers 2 and 3, iNAV images were significantly better (*p* = 0.0078 and *p* = 0.0088 respectively). (Image quality 1: Non-diagnostic, 2: Acceptable, 3: Good, 4 Full Diagnostic)

Figure 4 shows the image quality scores for each expert observer separately. For Expert 1 there was no statistically significant difference between image quality obtained with the dNAV and iNAV images (*p* = 1), while for the other two experts, iNAV images were significantly better than dNAV images (*p* = 0.0078 and *p* = 0.0088 respectively). Overall, average image quality for the iNAV images was 3.1 ± 1.0, while for dNAV images was 2.6 ± 1.0 (*p* = 0.0012). For both the assessment of iNAV images (*α* = 0.38) and dNAV images (*α* = 0.55), inter-rater reliability was found to be inconclusive (i.e., *α* below the cutoff criteria). For all reviewers, the presence of motion-induced artefacts was the most common reason for dNAV-based images not to be classified as full diagnostic, whilst motion blurring was the most common reason for image quality degradation with the water/fat iNAV-based approach.

**Fig. 5:**
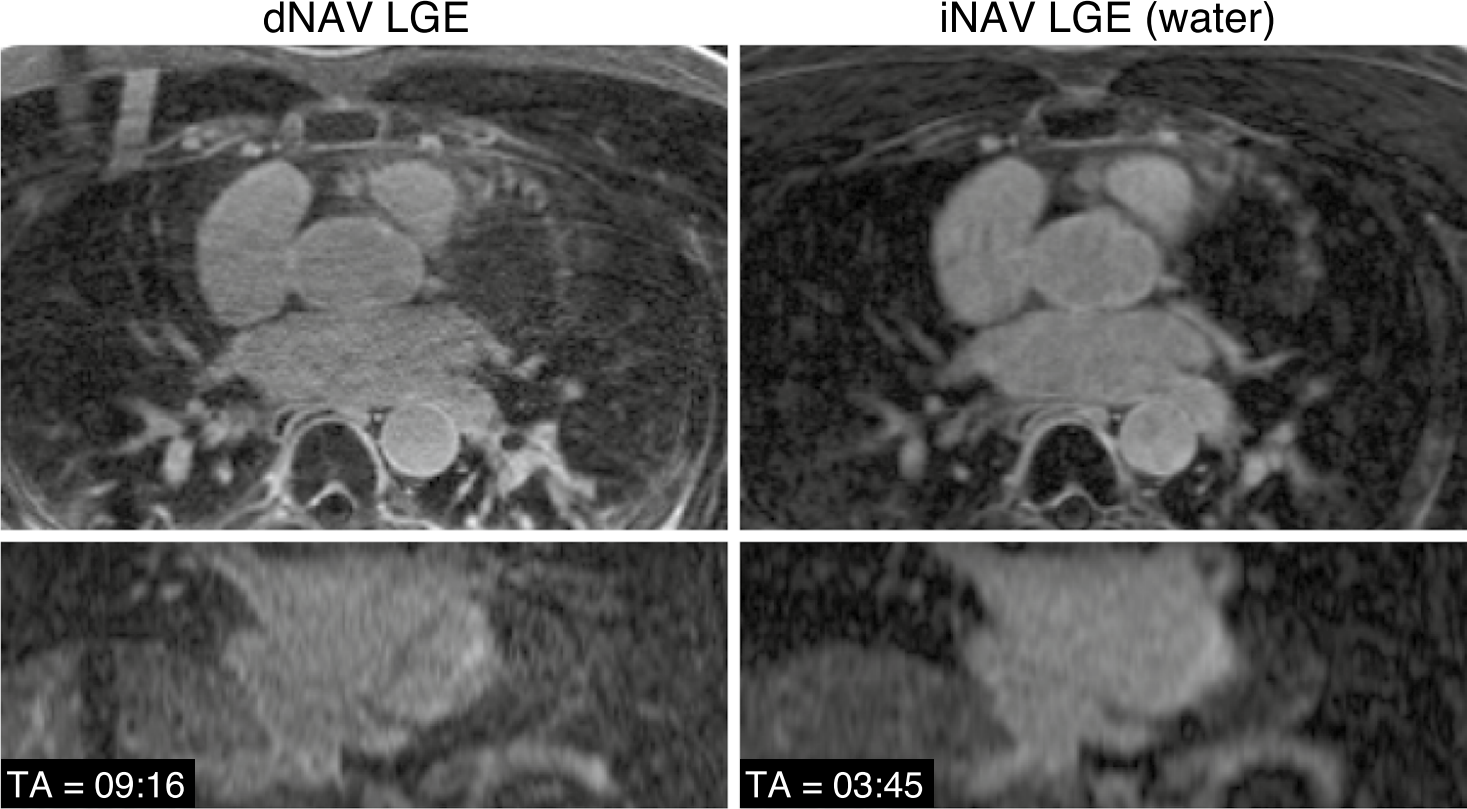
Visual comparison of 3D LGE images acquired with the conventional dNAV approach and the iNAV-based approach for a representative patient. Motion-induced artefacts in the dNAV-based images prevented this image from being used for diagnosis. Acquisition times (TA) are expressed as min:s

LGE presence was separately assessed by two experts. Both of them deemed iNAV images as inconclusive less frequently than with dNAV images. Indeed, the first expert rated 4 dNAV and 2 iNAV images as inconclusive; while the second expert found 7 dNAV and 4 iNAV images inconclusive. For both dNAV and iNAV images inter-rater reliability was found to be inconclusive regarding fibrosis detection, with *α* = 0.30 for dNAV images and *α* = 0.31 for iNAV images. A summary of the assessment of presence of fibrosis in the images can be found in Table 2. An example case where dNAV-based images resulted in an inconclusive diagnosis is shown in Fig. 5, where respiratory-induced artefacts rendered the image non-diagnostic.

**Table 2:** Detection of fibrosis

	dNAV-based images		iNAV-based images	
	Reader 1	Reader 2	Reader 1	Reader 2
Absent with confidence	4	3	3	2
Present with confidence	10	8	13	12
Inconclusive	4	7	2	4

Detection of fibrosis performed by two expert readers (Reader 1 with > 3 years of experience, Reader 2 with > 10 years of experience in cardiac MRI)

**Fig. 6:**
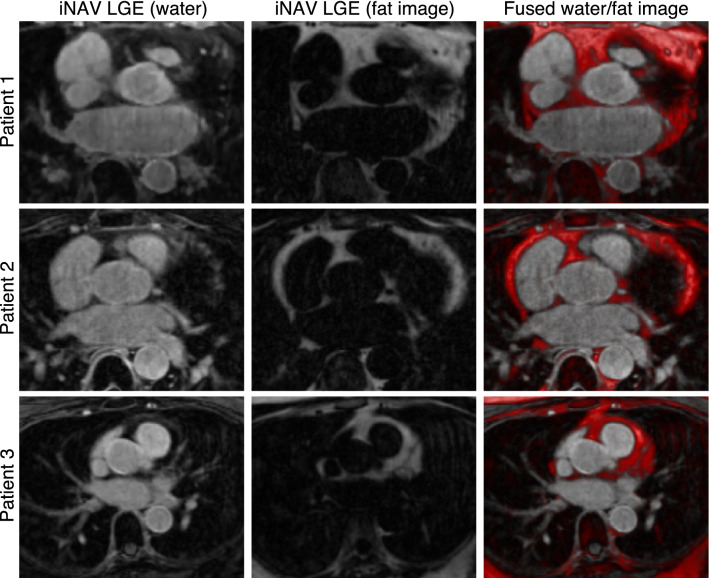
Proposed iNAV-based water/fat LGE atrial imaging protocol for three representative patients showing an example water LGE image, fat image, and a fused version of both (with fat depicted in red). The proposed imaging method provides a fully co-registered complementary fat image that can be used to quantify peri-atrial fat

Figure 6 shows example water/fat iNAV-based atrial LGE images for three representative patients, including the atrial LGE image (water), the corresponding fat image and a fused image. The water/fat iNAV-based approach produces a fully co-registered complementary fat image that may be used for characterization of peri-atrial fat.

**Fig. 7:**
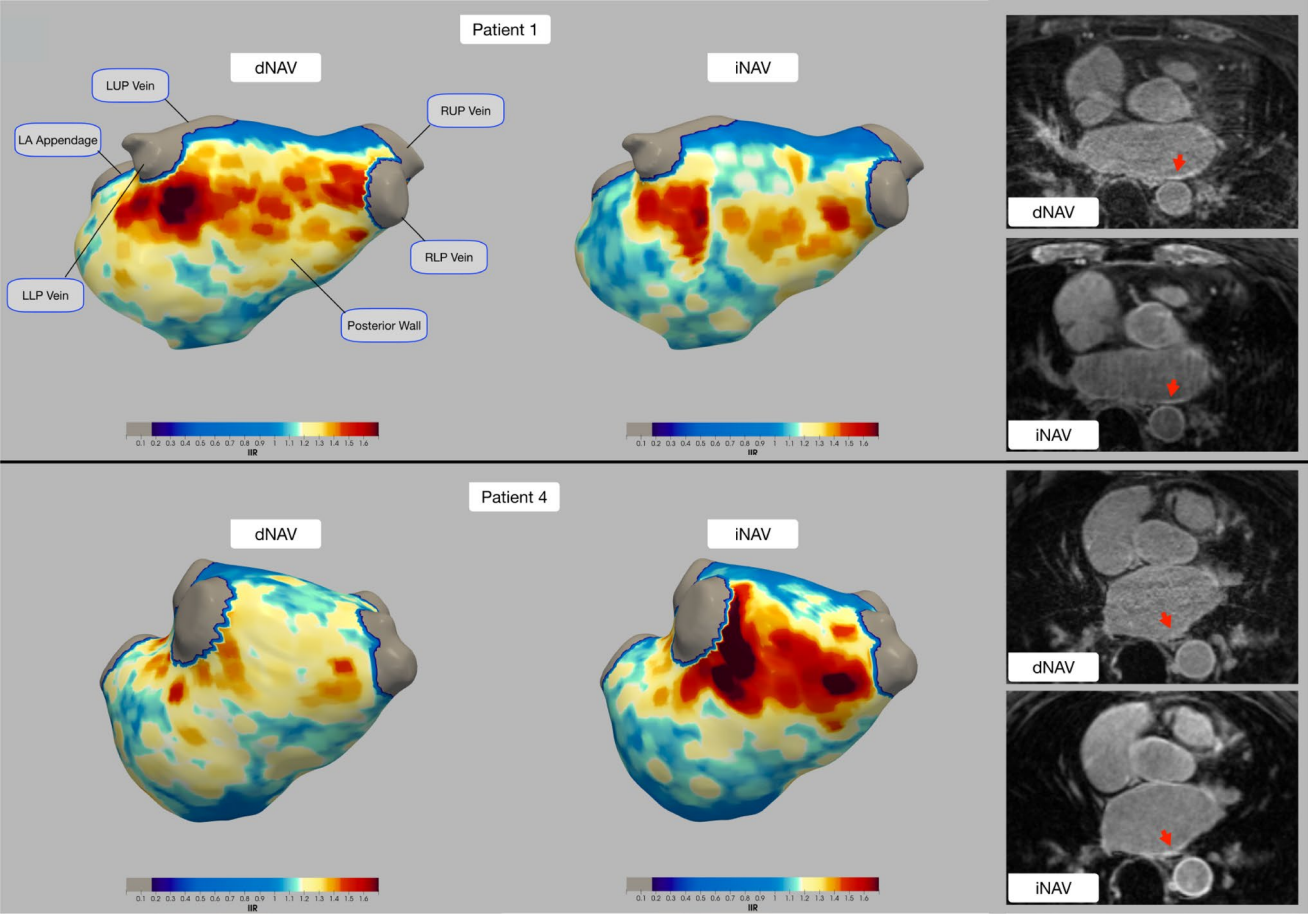
Atrial shells for two representative patients showing 3D visualization of fibrosis from dNAV and iNAV images, with fibrotic areas depicted in red. Example slices for each patient are also displayed, where fibrotic areas are indicated by red arrows. Similar scar patterns are seen between the dNAV and iNAV-based images; however, differences in contrast due to the difference in acquisition times post contrast administration result in some differences in the scan patterns observed in the rendered 3D images. *RUP* right upper pulmonary; *LUP* left upper pulmonary; *RLP* right lower pulmonary; *LLP* left lower pulmonary; *LA* left atrial

Despite iNAV-based images being acquired starting ~ 15 min after the conventional dNAV-based images (34.9 ± 5.9 min after administration of contrast agent), they resulted in overall good image quality and enabled good depiction of the atria and segmentation of atrial scar, as can be observed in the atrial shells in Fig. 7.

## Discussion

In this study we have demonstrated the feasibility of a highly efficient framework for 3D water/fat LGE atrial wall imaging in about 5 min. Compared to conventional atrial LGE imaging, which uses diaphragmatic navigator gating for respiratory motion compensation, this approach relies on coronal iNAVs that enable 100% scan efficiency, resulting in a predictable scan time that only depends on the subjects’ heart rate and volumetric coverage required. In addition, the use of an undersampled variable density trajectory enabled further reduction of total scan time, without adversely affecting image quality.

Visual scoring showed that iNAV images achieve equal or superior image quality compared to dNAV images, moreover, the iNAV-based method achieved a significantly shorter scan time, with a 59% reduction compared to the conventional method. Furthermore, with the iNAV-based water/fat approach two potential sources of image artefacts were minimized: no inflow artefacts in the pulmonary vein were observed, and fat suppression was improved by the water/fat separation approach. While these two sources of artefacts have been previously addressed by techniques that include modifying the acquisition sequence to minimize inflow artefact [12, 15, 16] or the use of water/fat sequences [18], such techniques did not address the issue of unpredictable and prolonged scan times, which remains a challenge for the clinical adoption of atrial LGE imaging.

It is worth noting that for the conventional dNAV-based images, the main reason for images to be rated as other than full diagnostic was because of motion-induced artefacts. Depending on their location in the image, these artefacts can obscure and/or reduce the confidence in the detection of fibrosis in the atrial wall, as they appear as high intensity structures which could be misinterpreted as areas of enhancement. On the other hand, for the iNAV-based method, residual blurring was the most common cause for impaired image quality. As iNAV-based images were always acquired after the dNAV-based images, longer TIs were required due to contrast agent washout, which limited the selection of an optimal atrial diastolic window for data acquisition and may have resulted in motion artifacts. Furthermore, residual motion in the anterior–posterior (AP) direction is currently not corrected for in a beat-to-beat basis. Approaches such as virtual 3D iNAVs that exploit autofocus to estimate beat-to-beat AP motion could alleviate this problem in future studies [33]. Nevertheless, in most cases such blurring did not affect the diagnostic quality of the resulting images.

Overall, in the cohort recruited for this study conventional atrial LGE imaging resulted in 22.2/38.8% inconclusive scans (Reader 1/Reader 2 respectively), which is similar to values reported elsewhere in the literature. The iNAV-based approach resulted instead in 11.1/22.2% inconclusive scans (Reader 1/Reader 2 respectively), showing promise for improved diagnostic quality of atrial LGE imaging despite suboptimal timing after contrast agent injection.

A moderate threefold acceleration (with elliptical shutter) was used in this study resulting in a scan time of about 5 min for a spatial resolution matching the atrial imaging protocol used routinely at our institution. Other techniques available for accelerated atrial LGE imaging have used similar acceleration factors [19, 34, 35] to achieve scan times of approximately 6 min. However, they use more complex compressed-sensing and respiratory-resolved image reconstruction algorithms, that require up to 60 min of computation time. In contrast, our method can achieve good image quality with an inline reconstruction that requires only a few minutes.

While in this study a moderate acceleration and spatial resolution were used, further acceleration in combination with regularized image reconstruction approaches could enable increased spatial resolution and potentially an improved depiction of atrial fibrosis. Moreover, further acceleration could enable the extension of the iNAV-based water/fat imaging protocol to a phase-sensitive inversion recovery (PSIR) [36] approach for improved contrast between areas of atrial wall enhancement and blood pool.

This study has a number of limitations. Patients recruited for this study underwent a clinically referred cardiac MRI protocol including a conventional 3D LGE imaging protocol, acquired always ~ 20 min after contrast agent administration, in order to produce optimal image quality and to avoid any possible impairment to the clinical data. The clinical protocol was then followed by the iNAV 3D water/fat LGE and therefore the timing after contrast injection for this sequence was suboptimal. Indeed, data acquisition with the iNAV-based sequence started ~ 15 min after the acquisition of conventional dNAV-based images, resulting in intrinsic differences in the washout of contrast agent. Indeed, the average selected TI for the dNAV-based images was 253.3 ± 32.5 ms, while for the iNAV-based images was 276.1 ± 24.8 ms (significantly longer, *p* = 0.008). This difference reflects changes in the overall contrast of the image due to contrast washout, and it is especially noticeable in areas such as the aortic wall and mitral valve, which often appeared hyper-enhanced in the iNAV-based water LGE images. It is worth noting that such enhancement is consistent with findings in the literature and likely reflect increased extracellular volume in the aortic wall and the intrinsic fibrous composition of the valves [18]. Moreover, such difference does not seem to affect the ability to detect atrial scar, and previous reproducibility studies have shown good agreement in scar detection for scans starting 20 and 30 min after contrast agent administration [9]. Further studies considering randomization of the order in which images are acquired will be performed to fully characterize the performance of both approaches. Furthermore, while the detection of areas of enhancement was comparable between the conventional dNAV and the iNAV-based methods, there was no available gold-standard for assessing the true distribution of fibrosis/scar in the atria to be used as reference. Future studies that compare fibrosis/scar depiction between the proposed method and a gold-standard voltage map acquired using 3D electroanatomic mapping are warranted.

In conclusion, accelerated motion-compensated water/fat atrial 3D LGE imaging allows for the depiction of atrial fibrosis and scar with good image quality, comparable to conventional diaphragmatic navigator-gated protocols but with a 59% shorter scan time of about 5 min.
